# Clinical Outcomes of Patients With Primary Membranous Nephropathy and Subnephrotic Proteinuria

**DOI:** 10.3389/fmed.2021.737700

**Published:** 2021-12-02

**Authors:** Peng He, Yang Zha, Jing Liu, Hanmin Wang, Lijie He

**Affiliations:** Department of Nephrology, Xijing Hospital, The Fourth Military Medical University, Xi'an, China

**Keywords:** primary membranous nephropathy, subnephrotic proteinuria, clinical relapse, kidney function progression, remission

## Abstract

**Objectives:** To update the information about the prognosis of patients with primary membranous nephropathy (MN) and subnephrotic proteinuria and identify the relevant predictors.

**Methods:** In total, 474 cases of biopsy-proven primary MN with at least 18 months of follow-up were reviewed to determine the outcomes of the subgroup of patients that presented with subnephrotic proteinuria. Clinical data included initial proteinuria and microhematuria, defined as the average proteinuria/microhematuria of the first 6 months during the course. Outcomes included partial remission (PR), complete remission (CR), nephrotic proteinuria progression, and kidney function progression, defined as ≥50% loss of kidney function or end-stage kidney disease.

**Results:** In total, 205 patients with primary MN and subnephrotic proteinuria at biopsy were eligible. During a median follow-up of 43 months, 200 (97.56%), 167 (81.46%), and 53 (25.85%) patients attained PR, CR, and nephrotic proteinuria progression, respectively. Only one patient (0.49%) progressed to the kidney function progression. By multivariate Cox hazards regression analyses, the initial proteinuria was identified as the independent predictor for PR, CR, and nephrotic proteinuria progression with adjusted hazard ratios (aHRs) of 0.67 (95% confidence interval, 0.56–0.80), 0.50 (95% CI, 0.40–0.63), and 2.97 (95% CI, 2.23–3.97), respectively. A higher level of initial microhematuria was also associated with an increased risk of nephrotic proteinuria progression. The corresponding aHR was 1.11 (95% CI, 1.05–1.17).

**Conclusion:** Among patients with primary MN and subnephrotic proteinuria, although the overall prognosis is excellent, dynamic detection and effective management of proteinuria remain important. In addition, initial microhematuria may be another predictor of nephrotic proteinuria progression.

## Introduction

Primary membranous nephropathy (MN), an autoimmune glomerular disease, is one of the most common causes of primary nephrotic syndrome in adults. Approximately 20% of patients with primary MN present with subnephrotic range proteinuria (24 h urinary protein excretion <3.5 g/d), however, 61% later develop nephrotic range proteinuria (24 h urinary protein excretion ≥3.5 g/d), usually within the first year (1–7). There is also evidence to support the long-term benefit of persistent subnephrotic proteinuria with renal survival of >80–90% at 10 years (8). Recently, the independent relationships between several clinical features and long-term renal function decline have been increasingly noted in the overall population of primary MN, e.g., age, male gender, increased proteinuria during the course, decreased estimated glomerular filtration rate (eGFR) on presentation, high levels of phospholipase A_2_ receptor (PLA2R) antibody after therapy, and C3 staining in the renal biopsy sample, etc. (2, 3, 5–7). However, among those with subnephrotic proteinuria, there was not sufficient data to describe the clinical prognosis and specific predictors.

Although current studies suggest these patients overall do well, the prognostic assessment of this subset of patients is controversial to some extent due to the lack of sufficient attention and data support. In the clinical practice, by virtue of the initial benign presentation and absence of specific predictive markers, some of these patients were not properly monitored and treated, putting them at the risk of developing nephrotic range protenuria and kidney function loss. To our knowledge, the study with the most sufficient sample size and follow-up was the cohort of Hladunewich et al. which indicated that, compared with patients with primary MN and persistent subnephrotic range proteinuria, those who subsequently attained nephrotic proteinuria showed a rate of kidney disease progression ~4 times faster. However, the only baseline feature with statistical significance between the two groups was the level of proteinuria; besides, a few years have passed (7).

Glomerular microhematuria is one of the most common symptoms of glomerulonephritis. Recently, it is considered as a biomarker of activity in IgAN, ANCA-associated vasculitis, or lupus nephritis, and some data indicated that persistent microhematuria in IgAN is related to a greater risk of kidney disease progression (9–17). In primary MN, microhematuria is not uncommon and accounts for ~50% of patients at presentation and 60% during the course (1, 2). However, based on the atypical nature of clinical characters and the absence of standardized methods to quantify (18, 19), little attention has been paid to the prognostic relevance of microhematuria in primary MN. Furthermore, Gutiérrez et al. suggested that the long-term outcomes in patients with biopsy-proven IgAN, isolated microhematuria, and minimal proteinuria at presentation are excellent (20). It is of great significance to systematically describe the prognosis of primary MN patients presenting with subnephrotic or minimal proteinuria and microhematuria at presentation.

In this retrospective cohort, we investigated the clinical course and outcomes among patients with primary MN and subneprotic range proteinuria. Longitudinal analyses were done to determine the corresponding prognostic factors and to quantify the strength of relevance.

## Methods

### Study Population

We included in the present study patients admitted to the Department of Nephrology, Xijing Hospital (Xi'an, China) between October 1, 2015, and June 30, 2019, with biopsy-proven primary MN and subnephrotic proteinuria (24 h urinary protein excretion <3.5 g/d) at baseline, established as the time of kidney biopsy. Additional inclusive criteria included: a baseline eGFR of >15 ml/min/1.73 m^2^, calculated according to the Chronic Kidney Disease Epidemiology Collaboration equation (21); at least 18 months of follow-up; and sufficient information on treatments and laboratory parameters for analyses. Patients with MN that associated with other diseases or exposures, such as infections, autoimmune diseases, malignancy, drugs/toxins, were diagnosed as secondary MN and excluded. Those with atypical MN (identified by kidney biopsy), or other concomitant glomerular diseases were also excluded. The study has been approved by the ethics committee of Xijing Hospital.

### Data Collected

Patients were collected retrospectively, and their medical history, clinical data, and standard laboratory parameters were documented. The follow-up data were last updated on 31 December 2020. Demographic characteristics included age, gender, body mass index (BMI), comorbidities, and smoking status at the time of kidney biopsy. Initial and follow-up variables involved the assessment of blood pressure (BP), serum creatinine, serum albumin, microscopic analysis of urinary sediment, as well as 24 h proteinuria excretion. As for the microhematuria, we only recorded the results of urine sediment analyses, mainly of glomerular derived erythrocytes (dysmorphic erythrocytes >70%). The titers of serum anti-PLA2R antibody were tested using indirect immunofluorescence assays (IIFAs) and reported according to the fluorescence intensities and dilutions (1:10, 1:100, 1:1000) of the serum samples. The intensities of PLA2R and C3 staining in pathological specimens (using the IIFAs) were standardly reported and recorded as -, +, ++, +++, or ++++. The exposure to immunosuppressive (IS) agents (including corticosteroids, tacrolimus, and cyclophosphamide), statin class medications, and BP medications, including the angiotensin-converting enzyme inhibitors (ACEIs) or angiotensin receptor blockers (ARBs), were reported as intent to treat regardless of the duration of exposure.

### Definitions and Outcomes

The follow-up time referred to the interval between kidney biopsy and the last outpatient visit, death, or end-stage renal disease (ESRD), whichever occurred first. The ESRD was defined by an eGFR value of <15 ml/min/1.73 m^2^ or need of chronic dialysis. Hypertension was defined by systolic BP ≥ 140 mmHg, or diastolic BP ≥ 90 mmHg, or taking BP medications. Additionally, a high level of serum anti-PLA2R antibody was defined by a titer of ≥1:100, and high intensity of PLA2R or C3 staining was defined as an intensity of ≥ ++ in the IIFAs. Due to the high variability of proteinuria and microhematuria assessment at a single time, initial proteinuria (initial microhematuria) referred to the average proteinuria (microhematuria) of the first 6-month block during follow-up. Initial persistent microhematuria was defined by an initial microhematuria of >5 red blood cell counts (RBCs)/high-power field (HPF).

As for patients with primary MN and subnephrotic proteinuria, partial remission (PR) was defined by a proteinuria value of <3.5 g/d plus a ≥50% reduction from its peak value, with stable kidney function. Complete remission (CR) was a proteinuria value of ≤ 0.3 g/d, along with normal serum albumin (≥3.5 g/dl) and stable kidney function. Nephrotic proteinuria progression was defined by an appearance of proteinuria ≥3.5 g/d. The primary outcomes were the PR, CR, and nephrotic proteinuria progression. The secondary outcome was kidney function progression, defined by a ≥50% decline in the eGFR or ESRD.

### Statistical Analysis

Metric data were summarized as mean with standard deviation (SD) or median with interquartile range (IQR) according to its distribution. Categorical data were expressed as numbers with percentages. The included participants were classified into three groups (T1, T2, and T3) in reference to tertiles of initial proteinuria. The comparisons between groups were done using tests for trend, involving Cochran-Armitage trend test or Spearman's rank correlation test as appropriate. Clinical characteristics for never nephrotic and nephrotic proteinuria progression groups were compared using Student's *t*-test for normally distributed variables, Wilcoxon-rank sum test for variables with skewed distributions, and the chi-squared test for categorical variables.

Cumulative incidence rates of PR, CR, and nephrotic proteinuria progression were plotted and compared using Kaplan-Meier analyses and Log-rank tests. Univariate and multivariate Cox hazards regression analyses were done to identify independent prognostic factors and yield hazard ratios (HRs) with 95% confidence intervals (CIs). The variables with *P* < 0.05 in univariate analyses were included in multivariate analyses. For all the analyses, complete case methods were adopted, for which each analysis was restricted to participants with complete data for all factors in models. A two-sided *P* < 0.05 was considered statistically significant. SPSS version 22.0 (IBM, Chicago, IL, USA) and Stata version 15.0 (Stata Corporation, College Station, TX, USA) were used for statistical analyses.

## Results

**Figure 1:**
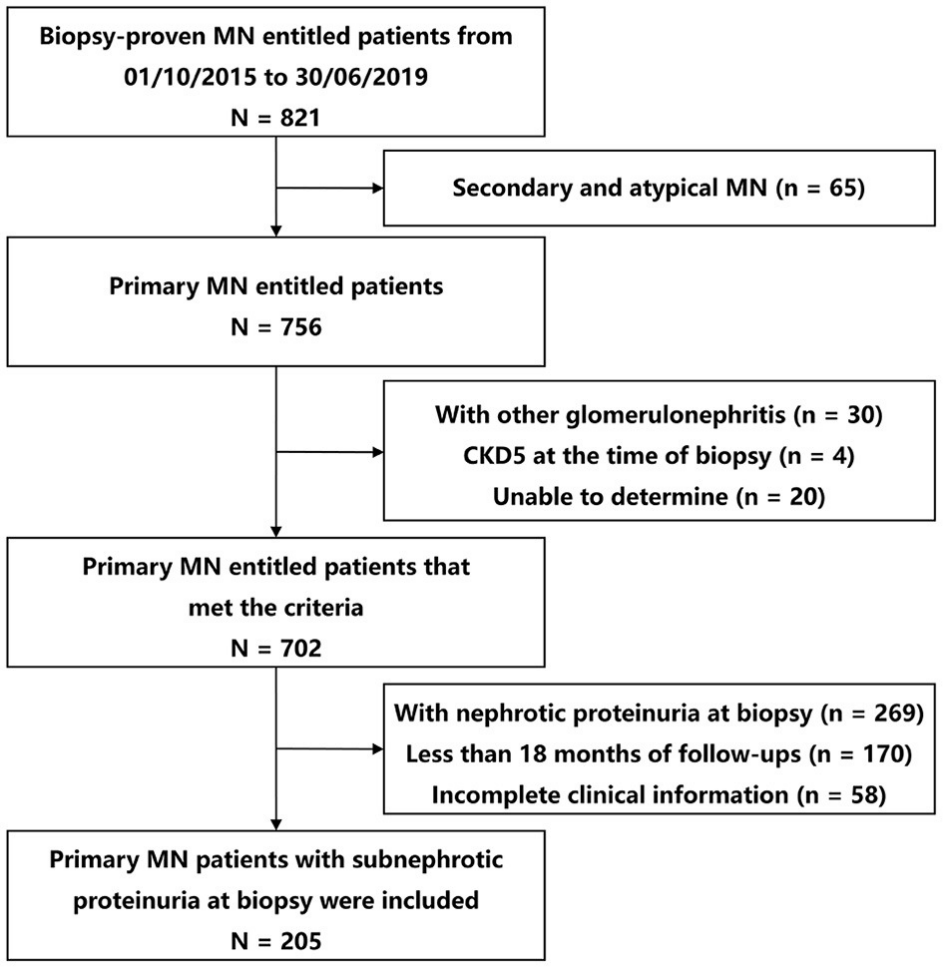
Study flow diagram of patient selection. MN, membranous nephropathy; CKD, chronic kidney disease.

In the entire cohort, among participants with primary MN, 27.12% (205 of 756) presented with subnephrotic range proteinuria, and preserved kidney function were collected (Figure 1). Their baseline and follow-up data are shown in Table 1. The median age was 48 years (IQR, 31–55 years), and 103 (50.24%) patients were males. The mean BMI was 24.12 kg/m^2^ (SD, 3.82 kg/m^2^). In total, 64 (31.22%) patients were hypertensive, 15 (7.32%) patients had diabetes, and 38 (18.54%) were smokers. All patients had normal kidney function [the mean serum creatinine was 0.85 mg/dl (SD, 0.16 mg/dl), and the median eGFR was 96.47 ml/min/1.73 m^2^ (SD, 19.08 ml/min/1.73 m^2^)]. The median proteinuria was 1.62 g/d (IQR, 0.90–2.22 g/d), median microhematuria was 3 RBCs/HPF (IQR, 1–8 RBCs/HPF), and the mean serum albumin was 3.22 g/dl (SD, 0.73 g/dl). Additionally, 73 (40.78%), 110 (65.87%), and 24 (13.79%) presented with a high level of serum anti-PLA2R antibody, high intensity of PLA2R staining, and high intensity of C3 staining, respectively. In the primary analysis, data were missing for 7.32, 12.68, 18.54, and 15.12% of patients for BMI, serum anti-PLA2R antibody, the intensity of PLA2R staining, and intensity of C3 staining, respectively.

**Table 1:** Clinical characteristics according to the tertiles of the initial proteinuria.

**Variable**	**Total**	**Initial proteinuria**			***P*-value**
		**T1 (<0.98 g/d)**	**T2 (0.98–1.56 g/d)**	**T3 (>1.56 g/d)**	
Patient No.	205	68	69	68	
**Baseline**					
Age (IQR), years	48 (31–55)	44 (28–55)	49 (33–54)	49 (34.5–57.5)	0.185
Females (*n*, %)	102 (49.76)	43 (63.24)	30 (43.48)	29 (42.65)	0.017
BMI, Mean ± SD, kg/m^2^ 190	24.12 ± 3.82	23.52 ± 3.88	24.19 ± 3.54	24.70 ± 3.96	0.052
Systolic BP, Mean ± SD, mmHg	121.40 ± 16.80	119.16 ± 19.40	120.74 ± 14.31	124.32 ± 16.16	0.052
Diastolic BP, Mean ± SD, mmHg	73.88 ± 12.00	73.01 ± 12.70	73.16 ± 11.95	75.47 ± 11.33	0.171
Hypertension (*n*, %)	64 (31.22)	20 (29.41)	20 (28.99)	24 (35.29)	0.460
Diabetes (*n*, %)	15 (7.32)	2 (2.94)	5 (7.25)	8 (11.76)	0.049
Smokers (*n*, %)	38 (18.54)	13 (19.12)	16 (23.19)	9 (13.24)	0.379
Serum creatinine, Mean ± SD, mg/dl	0.85 ± 0.16	0.83 ± 0.18	0.89 ± 0.15	0.83 ± 0.15	0.756
EGFR, Mean ± SD, ml/min/1.73 m^2^	96.47 ± 19.08	96.82 ± 17.59	94.05 ± 19.71	98.57 ± 19.84	0.551
Serum albumin, Mean ± SD, g/dl	3.22 ± 0.73	3.41 ± 0.71	3.31 ± 0.75	2.94 ± 0.64	<0.001
Proteinuria (IQR), g/d	1.62 (0.90–2.22)	0.88 (0.42–1.31)	1.62 (1.05–2.30)	2.09 (1.69–2.78)	<0.001
Microhematuria (IQR), RBCs/HPF	3 (1–8)	3 (2–9)	3 (1–8)	3 (1–6)	0.167
High level of serum anti-PLA2R antibody (*n*, %) 179	73 (40.78)	20 (32.79)	23 (37.70)	30 (52.63)	0.030
High intensity of IF-PLA2R staining (*n*, %) 167	110 (65.87)	35 (62.50)	41 (70.69)	34 (64.15)	0.844
High intensity of IF-C3 staining (*n*, %) 174	24 (13.79)	6 (10.17)	9 (15.00)	9 (16.36)	0.336
**Follow-up**					
Initial proteinuria (IQR), g/d	1.26 (0.76–1.81)	0.64 (0.52–0.75)	1.26 (1.13–1.36)	2.16 (1.81–2.77)	<0.001
Initial microhematuria (IQR), RBCs/HPF	3.00 (2.00–5.20)	2.73 (2.00–6.40)	3.20 (2.00–4.50)	3.07 (1.50–6.05)	0.962
Follow-up duration (IQR), months	43 (34–53)	38.5 (29–49)	46 (38–54)	42.5 (35.5–53)	0.142
Treatment with ACEI/ARB (*n*, %)	179 (87.32)	60 (88.24)	61 (88.41)	58 (58.29)	0.607
Treatment with statin (*n*, %)	125 (60.98)	33 (48.53)	41 (59.42)	51 (75.00)	0.002
Treatment with corticosteroids (*n*, %)	132 (64.39)	37 (54.41)	49 (71.01)	46 (67.65)	0.108
Treatment with tacrolimus (*n*, %)	93 (45.37)	30 (44.12)	39 (56.52)	24 (35.29)	0.303
Treatment with cyclophosphamide (*n*, %)	56 (27.32)	10 (14.71)	18 (26.09)	28 (41.18)	0.001
**Outcomes (** ***n*** **, %)**					
Partial remission	200 (97.56)	68 (100)	68 (98.55)	64 (94.12)	0.027
Complete remission	167 (81.46)	64 (94.12)	55 (79.71)	48 (70.59)	<0.001
Nephrotic proteinuria progression	53 (25.85)	7 (10.29)	15 (21.74)	31 (45.59)	<0.001
40% decline in the eGFR	6 (2.93)	2 (2.94)	1 (1.45)	3 (4.41)	0.612
50% decline in the eGFR	1 (0.49)	0	0	1 (1.47)	0.220
End-stage renal disease	1 (0.49)	0	0	1 (1.47)	0.220

*SD, standard deviation; IQR, interquartile range; BMI, body mass index; BP, blood pressure; eGFR, estimated glomerular filtration rate; RBCs, red blood cell counts; HPF, high-power field; PLA2R, phospholipase A2 receptor; IF, immunofuorescence; ACEI, angiotensin converting enzyme inhibitor; ARB, angiotensin receptor blockers; MN, membranous nephropathy*.

The median follow-up time was 43 months (IQR, 34–53 months). The medians of initial proteinuria and microhematuria were 1.26 g/d (IQR, 0.76–1.81 g/d) and 3.00 RBCs/HPF (IQR, 2.00–5.20 RBCs/HPF), respectively. A total of 179 (87.32%) patients received treatment with either ACEIs or ARBs. In total, 125 (60.98%) patients received statin class medications. Additionally, 132 (64.39%) patients were treated with corticosteroids and/or other IS agents during follow-up.

**Figure 2:**
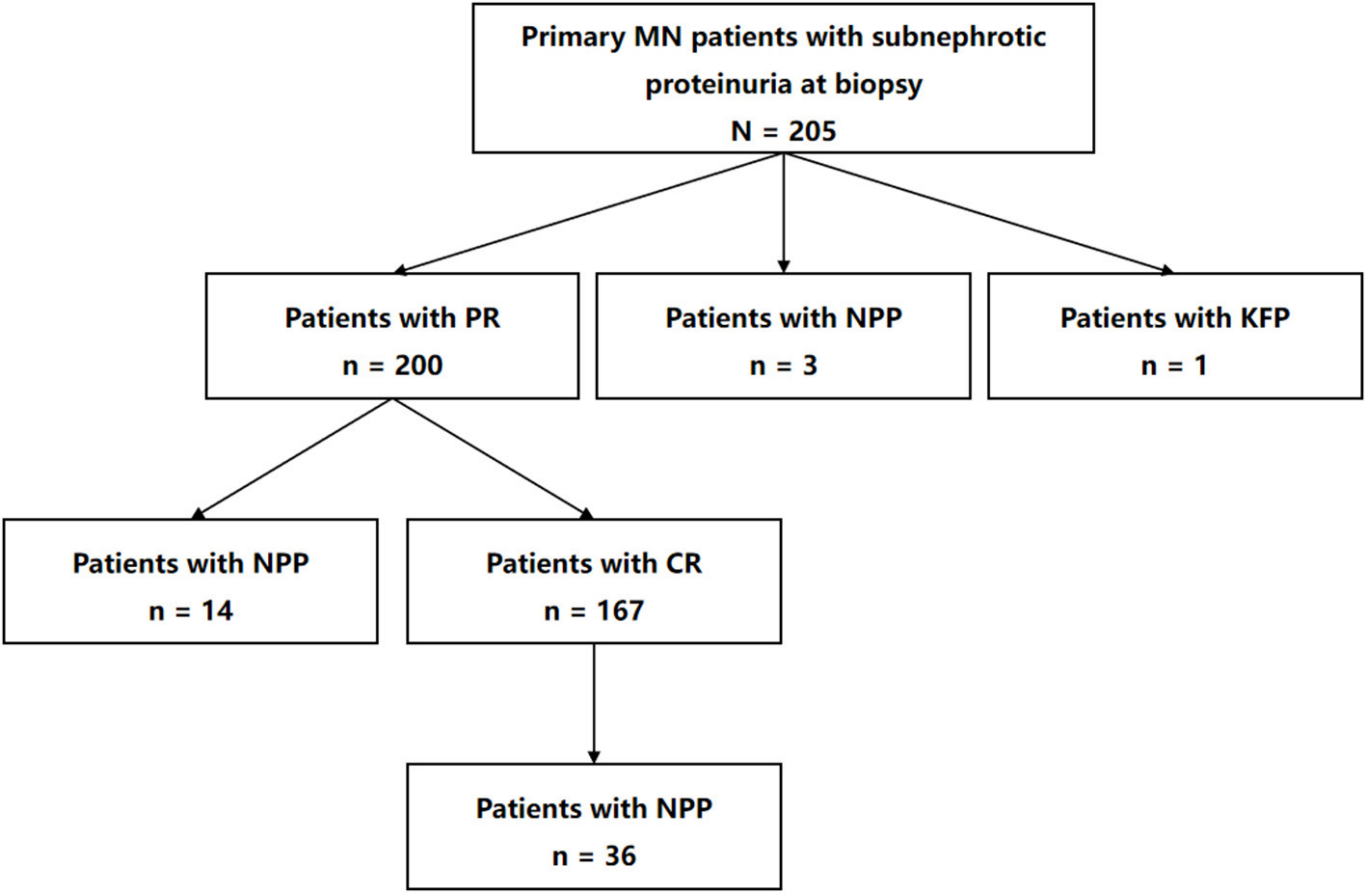
Flow diagram of numbers and sequences of the participants that reached various outcomes in the study. MN, membranous nephropathy; PR, partial remission; NPP, nephrotic proteinuria progression; KFP, kidney function progression; CR, complete remission.

The numbers and sequences of the eligible patients that reached various outcomes are presented in Figure 2. During our observational period, 97.56% of the entire cohort (200 of 205) achieved PR. The median time from kidney biopsy to PR was 5 months (IQR, 3–8 months). Among patients that attained PR, 83.5% (167 of 200) achieved CR in a median of 12 months (IQR, 8–21 months) after kidney biopsy. To sum up, 25.85% (53 of 205) of the entire cohort suffered nephrotic proteinuria progression in a median of 38 months (IQR, 22–49 months), including 36 patients who suffered nephrotic proteinuria progression after reaching PR and CR, 14 after PR, and three without remission. Additionally, only 2.93% (six of 205) patients suffered ≥40% loss of kidney function, and 0.49% (1 of 205) progressed to reach the pre-defined kidney function progression endpoint.

The eligible patients were classified according to the tertiles of initial proteinuria (0.96 and 1.53 g/d) (Table 1). From T1 to T3, those with higher levels of initial proteinuria were more likely to be females, Diabetes, and received statin or cyclophosphamide therapy. As initial proteinuria increased, the level of serum albumin decreased, whereas the level of serum anti-PLA2R antibody increased. As for the outcomes, from T1 to T3, the proportions of PR and CR decreased, whereas the proportion of nephrotic proteinuria progression increased (*P* < 0.05).

### Partial Remission

**Figure 3:**
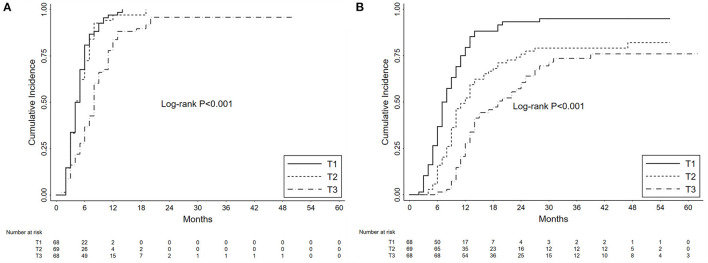
Kaplan-Meier curves depict the cumulative probabilities of partial remission **(A)** and complete remission **(B)** for patients with primary membranous nephropathy and subnephrotic range proteinuria. The patients were grouped by the tertiles (T1 vs. T2 vs. T3) of initial proteinuria. The time zero was a kidney biopsy. Log-rank tests were used for the comparison between groups.

In our cohort, the 1-year and 3-year cumulative probabilities of PR were 92.68 and 98.54%, respectively. As shown in Figure 3A, from T1 to T3 groups, the 1-year cumulative probabilities of PR were 97.06, 97.10 and 83.82%, respectively (*P* < 0.001). The results of Cox hazards regression analyses are summarized in Table 2 and Supplementary Table 1. In the multivariate model, the initial proteinuria was an independent prognostic factor of PR with a HR of 0.67 (95% CI, 0.56–0.86; *P* < 0.001).

**Table 2:** Univariate and multivariate analyses of independent prognostic factors of partial remission.

**Factor**	**Univariate analysis**		**Multivariate analysis**	
	**Hazard ratio (95% CI)**	***P*-value**	**Hazard ratio (95% CI)**	***P*-value**
Serum albumin (per 1 g/dl increase)	1.41 (1.17–1.70)	<0.001	1.20 (0.99–1.46)	0.063
Initial proteinuria (per 1 g/d increase)	0.64 (0.54–0.76)	<0.001	0.67 (0.56–0.80)	<0.001

*CI, confidence interval*.

### Complete Remission

During the follow-up, the 1-year and 3-year cumulative probabilities of CR were 53.17 and 82.41%, respectively. As shown in Figure 3B, from T1 to T3 groups, the 1-year cumulative probabilities of CR were 79.41, 52.17, and 27.94%, respectively, and the 3-year cumulative probabilities were 94.96, 79.10, and 73.57%, respectively (*P* < 0.001). The results of Cox hazards regression analyses are presented in Table 3 and Supplementary Table 2. In the multivariate model, the initial proteinuria and treatment with corticosteroids were independent prognostic factors of CR with HRs of 0.50 (95% CI, 0.40–0.63; *P* < 0.001) and 2.02 (95% CI, 1.43–2.85; *P* < 0.001), respectively.

**Table 3:** Univariate and multivariate analyses of independent prognostic factors of complete remission.

**Factor**	**Univariate analysis**		**Multivariate analysis**	
	**Hazard ratio (95% CI)**	***P*-value**	**Hazard ratio (95% CI)**	***P*-value**
Age (per 1 year increase)	0.99 (0.98–1.00)	0.037	0.99 (0.98–1.00)	0.194
Initial proteinuria (per 1 g/d increase)	0.56 (0.45–0.70)	<0.001	0.50 (0.40–0.63)	<0.001
Treatment with corticosteroids (yes vs. no)	1.57 (1.13–2.18)	0.008	2.02 (1.43–2.85)	<0.001

*CI, confidence interval*.

### Nephrotic Proteinuria Progression

Clinical characteristics of patients in the never nephrotic and nephrotic proteinuria progression groups are summarized in Table 4. Compared with patients in the never nephrotic group, those in the nephrotic proteinuria progression group showed higher levels of initial proteinuria [1.78 (IQR, 1.21–2.59) vs. 1.16 (IQR, 0.67–1.52) g/d; *P* < 0.001] and initial microhematuria [4.20 (IQR, 3.00–7.75) vs. 2.67 (IQR, 1.64–4.59) RBCs/HPF; *P* < 0.001], and a lower level of serum albumin [3.01 (SD, 0.73) vs. 3.29 (SD, 0.72) g/dl; *P* = 0.013].

**Table 4:** Clinical characteristics between never nephrotic and nephrotic proteinuria progression groups.

**Variable**	**Never nephrotic**	**Nephrotic progression**	***P*-value**
Patients No.	152	53	
Age (IQR), years	49 (33–55)	45 (27–54)	0.144
Females, *n* (%)	82 (53.95)	20 (37.74)	0.042
BMI, Mean±SD, kg/m^2^	23.92 ± 3.68	24.74 ± 4.18	0.197
Hypertension, *n* (%)	51 (33.55)	13 (24.53)	0.222
Diabetes, *n* (%)	14 (9.21)	1 (1.89)	0.122
Smokers, *n* (%)	28 (18.42)	10 (18.87)	0.943
eGFR, Mean ± SD, ml/min/1.73 m^2^	95.20 ± 18.39	100.12 ± 20.67	0.106
Serum albumin, Mean ± SD, g/dl	3.29 ± 0.72	3.01 ± 0.73	0.013
Initial proteinuria (IQR), g/d	1.16 (0.67–1.52)	1.78 (1.21–2.59)	<0.001
Initial microhematuria (IQR), RBCs/HPF	2.67 (1.64–4.59)	4.20 (3.00–7.75)	<0.001
High level of serum anti-PLA2R antibody, *n* (%)	51 (38.35)	22 (47.83)	0.259
High intensity of IF-PLA2R staining, *n* (%)	81 (65.85)	29 (65.91)	0.995
High intensity of IF-C3 staining, *n* (%)	17 (12.98)	7 (16.28)	0.586
ACEI/ARB, *n* (%)	134 (88.16)	45 (84.91)	0.540
Statin, *n* (%)	89 (58.55)	36 (67.92)	0.228
Corticosteroids, *n* (%)	97 (63.82)	35 (66.04)	0.771
Tacrolimus, *n* (%)	64 (42.11)	29 (54.72)	0.112
Cyclophosphamide, *n* (%)	42 (27.63)	14 (26.42)	0.864

*SD, standard deviation; IQR, interquartile range; BMI, body mass index; BP, blood pressure; eGFR, estimated glomerular filtration rate; RBCs, red blood cell counts; HPF, high-power field; PLA2R, phospholipase A2 receptor; IF, immunofuorescence; ACEI, angiotensin converting enzyme inhibitor; ARB, angiotensin receptor blockers*.

**Figure 4:**
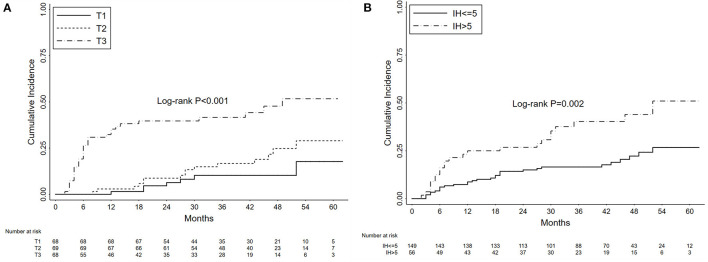
Kaplan-Meier curves depicting the cumulative probabilities of nephrotic proteinuria progression for patients with primary membranous nephropathy and subnephrotic range proteinuria. The patients were grouped by the tertiles of initial proteinuria (T1 vs. T2 vs. T3) **(A)** and the levels of initial hematuria (IH, >5 vs. ≤ 5 RBCs/HPF) **(B)**. The time zero was kidney biopsy. Log-rank tests were used for the comparison between groups.

The cumulative probabilities of nephrotic proteinuria progression were 13.17, 22.96, and 33.15% after 1, 3, and 5 years, respectively. As shown in Figure 4A, the 3-year cumulative probabilities of nephrotic proteinuria progression were 10.22, 16.65, and 41.59%, respectively, and the 5-year cumulative probabilities were 17.70, 29.00, and 51.75%, respectively, in the T1, T2, and T3 groups (*P* < 0.001). In Figure 4B, the 3-year and 5-year cumulative probabilities of nephrotic proteinuria progression were 16.53 and 26.67%, respectively, in the no persistent microhematuria group, and 40.27 and 51.00%, respectively, in the initial persistent microhematuria group (*P* = 0.002).

The results of Cox hazards regression analyses are summarized in Table 5 and Supplementary Table 3. In the multivariate model, the female gender, initial proteinuria, and initial microhematuria were identified as independent prognostic factors of nephrotic proteinuria progression. The corresponding adjusted HRs were 0.49 (95%CI, 0.28–0.87; *P* = 0.016), 2.97 (95%CI, 2.23–3.97; *P* < 0.001), and 1.11 (95%CI, 1.05–1.17; *P* < 0.001).

**Table 5:** Univariate and multivariate analyses of independent prognostic factors of nephrotic proteinuria progression.

**Factor**	**Univariate analysis**		**Multivariate analysis**	
	**Hazard ratio (95% CI)**	***P*-value**	**Hazard ratio (95% CI)**	***P*-value**
Female gender (vs. male gender)	0.54 (0.31–0.94)	0.029	0.49 (0.28–0.87)	0.016
Serum albumin (per 1 g/dl increase)	0.62 (0.43–0.90)	0.011	0.84 (0.57–1.25)	0.392
Initial proteinuria (per 1 g/d increase)	2.93 (2.22–3.88)	<0.001	2.97 (2.23–3.97)	<0.001
Initial microhematuria (per 1 RBC/HPF increase)	1.08 (1.03–1.13)	0.001	1.11 (1.05–1.17)	<0.001

*CI, confidence interval; RBC, red blood cell count; HPF, high-power field*.

### Kidney Function Progression

During the course, 2.93% (six of 205) patients suffered a 40% loss of kidney function. Their main clinical characteristics are summarized in Table 6. Four patients (Table 6, patients 1, 3, 4, 6) showed the initial proteinuria values of >1.00 g/d, and four patients (Table 6, patients 1, 3, 4, 5) showed the initial microhematuria values of >5.00 RBCs/HPF. During follow-up, five patients (Table 6, patients 1, 2, 4, 5, 6) were treated with either ACEIs or ARBs, and 3 (Table 6, patients 3, 4, 5) were treated with IS agents. Final proteinuria >1.00 g/d was observed in three patient (Table 6, patients 3, 4, 6). In the end, four patients (Table 6, patients 1, 2, 4, 5) reached PR, and two patients (Table 6, patients 1, 4) suffered nephrotic proteinuria progression.

**Table 6:** Clinical characteristics of patients with 40% loss in the kidney function.

**Variable**	**Patient 1**	**Patient 2**	**Patient 3**	**Patient 4**	**Patient 5**	**Patient 6**
Age (years)	68	50	61	67	55	59
Gender	Female	Female	Male	Male	Female	Female
Baseline serum albumin (g/dl)	3.62	3.83	3.24	2.49	3.16	2.11
Baseline eGFR (ml/min/1.73 m^2^)	69.4	80.1	92.1	78	94.0	90.0
Initial proteinuria (g/d)	1.62	0.23	2.50	1.37	0.79	2.07
Initial microhematuria (RBCs/HPF)	5.5	2.4	5.2	11	9.5	3
Final serum albumin (g/dl)	3.72	4.52	3.51	3.71	4.24	2.32
Final eGFR (ml/min/1.73 m^2^)	35.5	50.4	51	39.1	52.6	13.6
Final proteinuria (g/24 h)	0.81	0.25	2.05	7.48	0.13	2.25
ACEI/ARB treatment	Yes	Yes	No	Yes	Yes	Yes
Immunosuppressive treatment	No	No	CS+TAC	CS+CTX	CS	No
Follow-up (months)	36	43	50	52	54	18

*EGFR, estimated glomerular filtration rate; RBCs, red blood cell counts; HPF, high-power field; ACEI, angiotensin converting enzyme inhibitor; ARB, angiotensin receptor blockers; CS, corticosteroids; TAC, tacrolimus; CTX, cyclophosphamide*.

Kidney function progression was observed in only one patient (Table 6, patient 6). She was a 59-year-old woman whose renal biopsy showed stage II in histological classification. Baseline renal function and blood pressure were normal. The initial proteinuria and microhematuria were 2.07 g/d and 3.00 RBCs/HPF, respectively. Despite the administration of BP medications, the levels of 24 h urine protein excretion were consistently higher than 2.00 g/d during follow-up. A total of 18 months after kidney biopsy, kidney function showed an irreversible decline to a serum creatinine value of 3.43 mg/dl (eGFR, 13.63 ml/min/1.73 m^2^).

## Discussion

Our results suggested that, among patients with primary MN and subnephrotic proteinuria at kidney biopsy, the initial proteinuria, defined as the mean proteinuria of the first 6 months during follow-up, was an independent prognostic factor of PR, CR, and nephrotic proteinuria progression. Moreover, a higher level of the initial microhematuria, defined as the mean microhematuria of the first 6 months during follow-up, was associated with an increased risk of nephrotic proteinuria progression. For all we know, this is the first study to evaluate the prognostic relevance of microhematuria in this type of patient.

As one of the most common causes of adult-onset primary nephrotic syndrome, primary MN is most typically depicted as being accompanied by nephrotic range proteinuria (1, 2, 5, 7). For those in the subnephrotic status, guidelines classified most of them as a low-risk group and recommended conservative treatment (22, 23). However, close follow-up of these patients remains important, as a significant proportion of the patients who present with low-level of proteinuria will evolve to nephrotic range proteinuria and then follow a course similar to the classic nephrotic at-presentation group (7). Overall, our results showed that the prognosis of this subset of patients was generally excellent. During a median follow-up of 43 months, 200 (97.56%), 167 (81.46%), and 53 (25.85%) patients reached PR, CR, and nephrotic proteinuria progression, respectively. The 1-year, 3-year, and 5-year cumulative incidences of nephrotic proteinuria progression were 13.17, 22.96, and 33.15%, respectively. Only one patient (0.49%) progressed to the kidney function progression, defined as ≥50% loss of kidney function or ESRD.

One of the primary findings of our study was that the initial proteinuria, referred to as the average proteinuria of the first 6 months during the course, was an independent predictor of PR, CR, or nephrotic proteinuria progression. In the multivariate analyses, the corresponding aHRs were 0.67 (95% CI, 0.56–0.80), 0.50 (95% CI, 0.40–0.63), and 2.97 (95% CI, 2.23–3.97), respectively. The result suggested that the dynamic detection and effective management of proteinuria contributed to improving the prognosis of this subset of patients. In the cohort of Hladunewich et al. the only distinguishing baseline feature between the never nephrotic group and the nephrotic post-presentation group was a higher level of proteinuria in the group that subsequently developed nephrotic syndrome [1.98 (IQR, 0.3–3.4) vs. 2.43 (IQR, 0.5–3.4) g/d]. Compared with the proteinuria at baseline, the advantage of initial proteinuria was that it not only avoided the bias and instability of single-point detection but also depicted the initial treatment response of proteinuria. It is reasonable to expect more large-scale and prospective studies to confirm the clinical implications of our findings.

Microscopic hematuria has been previously considered as an indicator for a future flare in patients with systematic lupus erythematosus (SLE). Ding et al. suggested that changes in urinary sediments, either isolated microscopic hematuria or accompanied by sterile pyuria, were related to the disease activity among patients with SLE (13). Rhee et al. suggested that cumulative duration of microscopic hematuria was a possible biomarker of subsequent nephritis relapse in ANCA-associated vasculitis (11). In our study, among patients with primary MN and subneprotic proteinuria at kidney biopsy, the initial microhematuria was an independent risk factor of nephrotic proteinuria progression, and the corresponding aHR was 1.11 (95% CI, 1.05–1.17) in the multivariate analysis. The result highlighted the prognostic value of microhematuria for the nephrotic proteinuria progression in those with primary MN and low-grade proteinuria. Though the underlying mechanism is unclear, multiple studies have suggested that persistent glomerular microhematuria might represent a continued “low-grade” activity of the underlying inflammatory process, which could stimulate kidney injury through the oxidative stress caused by the release of hemoglobin and iron from broken RBCs into renal tubular cells (9, 17, 18, 24–27). Additionally, more studies are needed to verify our findings and elucidate the prognostic relevance of microhematuria among this subset of patients. It could contribute to more reasonable monitoring and guidance of clinical medication use in this group of primary MN patients.

Our study also had several limitations. First, it was a single-center retrospective cohort accomplished with a review of medical records. The interpretation might be biased owing to selection error. Second, we did not make regression analyses because the number of patients who developed kidney function progression was relatively small. Even if we did, an excessively small number of outcome events could increase the risk of an unstable conclusion. So the influence of some confounding factors on the results could not be excluded. Furthermore, there was no standardized regimen for induction and maintenance therapy, and the treatment decisions were totally dependent on the preference of individual physicians. Therefore, these fundamental restrictions could not be avoided in the evaluation of the effect of each treatment. Additionally, the tests for serum anti-PLA2R antibody titers were conducted using the IIFAs, which enable only semi-quantitative measurement of the serum anti-PLA2R antibody. For follow-up research and monitoring, enzyme-linked immunosorbent assay (ELISA) is more suitable. However, before august 2017, the ELISA was not done in our nephrology laboratory.

In summary, our data indicated that primary MN patients presenting with subnephrotic proteinuria overall had a benign prognosis. During our follow-up period, ~80% of patients achieved complete remission of proteinuria, and only ~20% of patients re-developed nephrotic range proteinuria. The initial proteinuria was an independent predictor of PR, CR, or nephrotic proteinuria progression. Besides, the initial microhematuria might be an additional indicator for the nephrotic proteinuria progression and provide reference indices for clinicians to monitor and manage this subset of primary MN patients.

## Data Availability Statement

The raw data supporting the conclusions of this article will be made available by the authors, without undue reservation.

## Ethics Statement

The studies involving human participants were reviewed and approved by the Ethics Committee of Xijing Hospital. Written informed consent for participation was not required for this study in accordance with the national legislation and the institutional requirements.

## Author Contributions

LH, PH, and HW designed the study, analyzed the data, and drafted the manuscript. PH, JL, and YZ collected and entered data. LH and HW contributed to data acquisition and interpretation. All authors read and approved the final manuscript.

## Funding

The study was supported by grants from the National Natural Science Foundation of China (No. 81770764, 81770669).

## Conflict of Interest

The authors declare that the research was conducted in the absence of any commercial or financial relationships that could be construed as a potential conflict of interest.

## Publisher's Note

All claims expressed in this article are solely those of the authors and do not necessarily represent those of their affiliated organizations, or those of the publisher, the editors and the reviewers. Any product that may be evaluated in this article, or claim that may be made by its manufacturer, is not guaranteed or endorsed by the publisher.
