# Supplementary material for: Clinical Outcomes of Patients With Primary Membranous Nephropathy and Subnephrotic Proteinuria
Source: Front Med (Lausanne). 2021 Dec 2;8:737700. doi: 10.3389/fmed.2021.737700 (PMC8674415; doi:10.3389/fmed.2021.737700)
Supplement: Supplementary file 1 [file Data_Sheet_1.PDF]

Clinical Outcomes of Patients with Primary Membranous Nephropathy and Subnephrotic Proteinuria

Peng He[1], Yang Zha[1], Jing Liu[1], Hanmin Wang[1], Lijie He[1,*].

[1]Department of Nephrology, Xijing Hospital, the Fourth Military Medical University, Xi'an, Shaan xi, 710032, China.

*Correspondence should be addressed to 43218204@qq.com (L.J.H.)

Supplement material

Table S1. Univariate and multivariate analyses of independent prognostic factors of partial remission

Table S2. Univariate and multivariate analyses of independent prognostic factors of complete remission

Table S3. Univariate and multivariate analyses of independent prognostic factors of nephrotic proteinuria progression

Table S1. Univariate and multivariate analyses of independent prognostic factors of partial remission

Factor	Univariate analysis		Multivariate analysis	
	Hazard ratio (95% CI)	*P* value	Hazard ratio (95% CI)	*P* value
Age	1.00 (0.99-1.01)	0.459		
Gender, female	1.03 (0.78-1.35)	0.861		
BMI	1.01 (0.97-1.04)	0.789		
Hypertension	1.05 (0.78-1.42)	0.744		
Diabetes	0.97 (0.57-1.64)	0.906		
Smokers	0.97 (0.68-1.39)	0.867		
EGFR	1.00 (0.99-1.01)	0.958		
Serum albumin	1.41 (1.17-1.70)	<0.001	1.20 (0.99-1.46)	0.063
Proteinuria	0.96 (0.81-1.13)	0.599		
Microhematuria	0.99 (0.97-1.00)	0.140		
High level of serum anti-PLA2R antibody	0.79 (0.59-1.08)	0.137		
High intensity of IF-PLA2R staining	1.13 (0.82-1.56)	0.462		
High intensity of IF-C3 staining	0.91 (0.59-1.41)	0.679		
Initial proteinuria	0.64 (0.54-0.76)	<0.001	0.67 (0.56-0.80)	<0.001
Initial microhematuria	0.97 (0.94-1.01)	0.126		
Treatment with ACEI/ARB	1.40 (0.90-2.18)	0.141		
Treatment with statin	0.90 (0.68-1.20)	0.477		
Treatment with corticosteroids	0.89 (0.66-1.18)	0.410		
Treatment with cyclophosphamide	0.86 (0.63-1.18)	0.357		
Treatment with tacrolimus	0.94 (0.70-1.25)	0.666		

MN, membranous nephropathy; CI, confidence interval; BMI, body mass index; eGFR, estimated glomerular filtration rate; PLA2R, phospholipase A2 receptor; IF, immunofuorescence; ACEI, angiotensin converting enzyme inhibitor; ARB, angiotensin receptor blockers.

Table S2. Univariate and multivariate analyses of independent prognostic factors of complete remission

Factor	Univariate analysis		Multivariate analysis	
	**Hazard ratio (95% CI)**	***P* value**	**Hazard ratio (95% CI)**	***P* value**
Age	0.99 (0.98-1.00)	0.037	0.99 (0.98-1.00)	0.194
Gender, female	1.16 (0.85-1.57)	0.347		
BMI	0.98 (0.94-1.02)	0.284		
Hypertension	0.96 (0.69-1.34)	0.828		
Diabetes	0.75 (0.41-1.38)	0.354		
Smokers	1.02 (0.68-1.51)	0.936		
EGFR	1.00 (0.99-1.01)	0.590		
Serum albumin	1.00 (0.81-1.23)	0.980		
Proteinuria	0.84 (0.70-1.00)	0.056		
Microhematuria	1.01 (0.99-1.02)	0.477		
High level of serum anti-PLA2R antibody	0.85 (0.61-1.18)	0.328		
High intensity of IF-PLA2R staining	1.08 (0.76-1.55)	0.659		
High intensity of IF-C3 staining	0.95 (0.59-1.52)	0.823		
Initial proteinuria	0.56 (0.45-0.70)	<0.001	0.50 (0.40-0.63)	<0.001
Initial microhematuria	1.00 (0.96-1.04)	0.964		
Treatment with ACEI/ARB	0.85 (0.53-1.34)	0.473		
Treatment with statin	0.88 (0.65-1.21)	0.438		
Treatment with corticosteroids	1.57 (1.13-2.18)	0.008	2.02 (1.43-2.85)	<0.001
Treatment with cyclophosphamide	1.07 (0.77-1.49)	0.704		
Treatment with tacrolimus	1.14 (0.84-1.54)	0.416		

MN, membranous nephropathy; CI, confidence interval; BMI, body mass index; eGFR, estimated glomerular filtration rate; PLA2R, phospholipase A2 receptor; IF, immunofuorescence; ACEI, angiotensin converting enzyme inhibitor; ARB, angiotensin receptor blockers.

Table S3. Univariate and multivariate analyses of independent prognostic factors of nephrotic proteinuria progression

Factor	Univariate analysis		Multivariate analysis	
	**Hazard ratio (95% CI)**	***P* value**	**Hazard ratio (95% CI)**	***P* value**
Age	0.99 (0.97-1.00)	0.129		
Gender, female	0.54 (0.31-0.94)	0.029	0.49 (0.28-0.87)	0.016
BMI	1.05 (0.98-1.13)	0.197		
Hypertension	0.70 (0.37-1.31)	0.262		
Diabetes	0.22 (0.03-1.62)	0.138		
Smokers	1.07 (0.54-2.12)	0.854		
EGFR	1.01 (1.00-1.03)	0.076		
Serum albumin	0.62 (0.43-0.90)	0.011	0.84 (0.57-1.25)	0.392
Proteinuria	1.37 (1.01-1.86)	0.043		
Microhematuria	1.02 (1.00-1.04)	0.058		
High level of serum anti-PLA2R antibody	1.21 (0.68-2.17)	0.520		
High intensity of IF-PLA2R staining	0.88 (0.47-1.65)	0.694		
High intensity of IF-C3 staining	1.10 (0.49-2.49)	0.811		
Initial proteinuria	2.93 (2.22-3.88)	<0.001	2.97 (2.23-3.97)	<0.001
Initial microhematuria	1.08 (1.03-1.13)	0.001	1.11 (1.05-1.17)	<0.001
Treatment with ACEI/ARB	0.74 (0.35-1.57)	0.432		
Treatment with statin	1.43 (0.80-2.55)	0.223		
Treatment with corticosteroids	0.99 (0.56-1.74)	0.960		
Treatment with cyclophosphamide	0.86 (0.47-1.59)	0.639		
Treatment with tacrolimus	1.59 (0.92-2.74)	0.094		

MN, membranous nephropathy; CI, confidence interval; BMI, body mass index; eGFR, estimated glomerular filtration rate; PLA2R, phospholipase A2 receptor; IF, immunofuorescence; ACEI, angiotensin converting enzyme inhibitor; ARB, angiotensin receptor blockers.
